# 22^nd^ AMN Congress in Bangkok, Thailand – Interview with Lt. Col. Assist. Prof. Panu Boontoterm

**DOI:** 10.25122/jml-2025-1009

**Published:** 2025-10

**Authors:** Stefana-Andrada Dobran, Alexandra Gherman

**Affiliations:** 1 RoNeuro Institute for Neurological Research and Diagnostic, Cluj-Napoca, Romania; 2 Sociology Department, Babes-Bolyai University, Cluj-Napoca, Romania

**Interviewee: Lt. Col. Assist. Prof. Panu Boontoterm**

**Interviewer: Stefana-Andrada Dobran**

Lt. Col. Assist. Prof. Panu Boontoterm is a double specialist in Neurological Surgery and Critical Care Medicine, working as a consultant surgeon and intensivist and the Head of the Surgical Intensive Unit at the Phramongkutklao Hospital in Bangkok, Thailand.

A dedicated member of the AMN for several years, he has participated at AMN Congresses as invited speaker, sharing his expertise, and, in 2025, has served as faculty for the NTSC Extended AMN intensives training course in Bangkok. Over the years, Lt. Col. Assist. Prof. Panu Boontoterm brought significant contributions to both clinical medicine and research.

**S.D.: Hello, dear Lt. Col. Assist. Prof. Panu Boontoterm. Welcome to the 22^nd^ AMN Congress here in Bangkok! What is your perspective on the AMN as a global player in the practice and science fields of TBI?**

P.B.: The Academy for Multidisciplinary Neurotraumatology has firmly established itself as a global leader in advancing both the science and clinical practice of traumatic brain injury. Its approach is uniquely positioned at the intersection of innovation, education, and collaboration. What makes AMN stand out is its commitment to fostering a truly multidisciplinary dialogue, bringing together neurosurgeons, neurologists, intensivists, rehabilitation experts, psychologists, and healthcare policymakers to comprehensively address the full spectrum of traumatic brain injury care. In a world where neurotrauma cases vary greatly by region, AMN, globally, is particularly impactful. Through its concrete training programs and research partnerships, the AMN is helping to enhance care while remaining sensitive to regional needs, especially in low- and middle-income countries where the burden of traumatic brain injury is disproportionately high and resources may be limited. Additionally, its engagement with global institutions such as the World Health Organization signals its growing influence in shaping neurotrauma policy at a systemic level. AMN is not only a scientific society, it's a catalyst for change in how we understand, manage, and teach neurotraumatology worldwide.


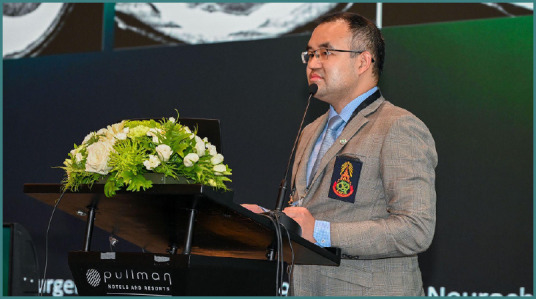


**S.D.: The 2025 Congress was preceded by the first edition of the NTSC Extended - AMN Intensives course, now at its first edition in Thailand. As a faculty member, please share with us your opinion regarding this educational event**.

P.B.: The Neurotrauma Treatment Simulation Course - AMN intensives in Thailand was an exemplary initiative that underscores the power of experiencing learning in neurotrauma education. As a faculty member, I found the course deeply rewarding both from a teaching and collaborative standpoint. The format, which combines cadaveric dissection with hands-on simulation stations and high-level expert discussions, offered participants a valuable opportunity to engage with real-world neurotrauma scenarios in a safe, controlled learning environment.

What truly impressed me was the diversity of participants from various countries and specialties who brought unique insights to neurotrauma care. The design of the event reflects the entire *chain of recovery* of neurotrauma patients from pre-hospital to long-term rehabilitation, which encourages holistic thinking and interprofessional problem solving. The integrative methodology is not only educational, but transformative, and I strongly believe it should become a replicable model for future regional and global teaching courses. The success of the Thailand edition set a strong precedent for expanding this initiative across Asia and underserved regions, as well as worldwide.


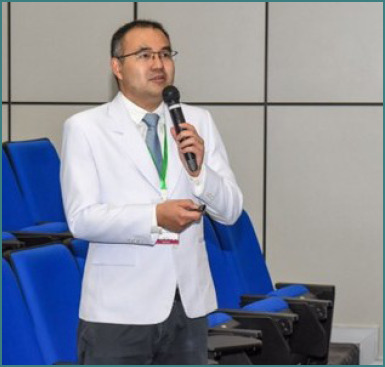


**S.D.: As a neurosurgeon, what is your intake related to the 2025 AMN Congress, and what would you foresee as future developments for the complex multidisciplinary domain of TBI?**

P.B.: The 2025 AMN Congress reinforces the central role of collaboration in addressing the intricate challenge of traumatic brain injury. As both a neurosurgeon and intensivist, I want everyone to see that this course is **moving beyond isolation, especially driving approaches toward** a more cohesive team-based model of care. The congress highlighted the latest [updates] in neuroprotection, neuroplasticity, data integration, and long-term rehabilitation, through the lens of multidisciplinary cooperation.


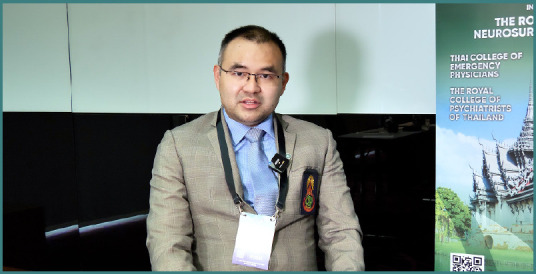


Looking forward, I anticipate several key developments that will shape the future of traumatic brain injury management. First, the integration of artificial intelligence and machine learning tools will enhance our ability to stratify patients, predict outcomes, and personalize team plans. Second, the expansion of centralized real-time data systems like the PRESENT registry will be instrumental in shaping policy and standard care across borders. Third, we must continue investing in simulation-based education, not just for medical professionals, but across the entire healthcare continuum, including emergency responders and rehabilitation teams. Ultimately, I believe the next evaluation of TBI care will center on interoperability between institutions, disciplines, and countries, and the AMN is in a unique position to lead that transformation to humanize the healthcare of traumatic brain injury patients.

**S.D.: How would you envision your future collaboration with the AMN academic environment?**

P.B.: I see my future within the AMN academic environment as one of active engagement and long-term commitment. I'm deeply inspired by the AMN mission and its dedication to multidisciplinary education, global inclusivity, and scientific excellence. I aim to contribute to several key areas by supporting the development and delivery of high-impact educational programs, participating in multicenter research initiatives, and helping to strengthen the regional training network, particularly in areas with limited access to neurotrauma expertise. I'm also eager to take on a mentorship role, guiding the next generation of neurosurgeons, intensivists, and neurorehabilitation professionals within the AMN framework. Building sustainable academic and clinical bridges between countries is a personal priority for me. I see the AMN as the ideal platform to facilitate connections worldwide. In the long term, I hope to contribute to the strategy vision of AMN, whether through curriculum design, registry development, and advocacy efforts at global health policy. Together with my colleagues, I believe we can raise the global standard of care in neurotraumatology and make a measurable difference in the life of patients worldwide.

